# Ultraviolet-C to mid-infrared supercontinuum generation in periodically poled lithium tantalate waveguides

**DOI:** 10.1038/s41377-026-02323-4

**Published:** 2026-05-26

**Authors:** Hongzhi Xiong, Xinmin Yao, Ming Zhang, Qingrui Yao, Huan Li, Zejie Yu, Gong Zhang, Liu Liu, Yaocheng Shi, Hon-ki Tsang, Daoxin Dai

**Affiliations:** 1 State Key Laboratory for Extreme Photonics and Instrumentation, Zhejiang Key Laboratory of Optoelectronic Information Technology, College of Optical Science and Engineering, International Research Center for Advanced Photonics, Zhejiang University, Zijingang Campus, Hangzhou, China; 2 Ningbo Global Innovation Center, Zhejiang University, Ningbo, China; 3 Department of Electronic Engineering, The Chinese University of Hong Kong, Hong Kong SAR, China; 4 China Jiliang University, Hangzhou, China

**Keywords:** Supercontinuum generation, Photonic devices

## Abstract

Supercontinuum generation makes use of the nonlinear optical effects arising from the interaction of light with the bound electronic states in crystal lattices and has many applications, especially in the ultraviolet for the direct probing of large-energy electronic transitions. However, supercontinuum from integrated waveguides has been limited to >330 nm in the ultraviolet-A band because of material dispersion and absorption. Here, we demonstrate unprecedented ultraviolet-C-to-mid-infrared supercontinuum on a chip, leveraging the exceptional transparency window and second-order nonlinearity of lithium tantalate (LT). A key innovation is the introduction of chirped periodically poled LT with submicron ferroelectric domains. Utilizing 3-wave-mixing processes under quasi-phase-matching conditions, we created the shortest ultraviolet wavelength ever reported from a chip—below 270 nm—while reaching 2400 nm in the mid-infrared, covering more than three octaves with just 100 pJ pulse energy on a chip for the first time. It’s the first on-chip supercontinuum fully covering the ultraviolet-A/B bands while extending into the ultraviolet-C band. This work establishes thin-film LT as a versatile platform for full-spectrum nonlinear photonics, opening new possibilities for integrated ultraviolet sources.

## Abstract (web summary)

Chirped periodically poled lithium tantalate waveguides enable on-chip supercontinuum spanning 270–2400 nm via quasi-phase-matched three-wave mixing, achieving ultraviolet-C to mid-infrared spectral coverage on a chip.

## Graphical abstract

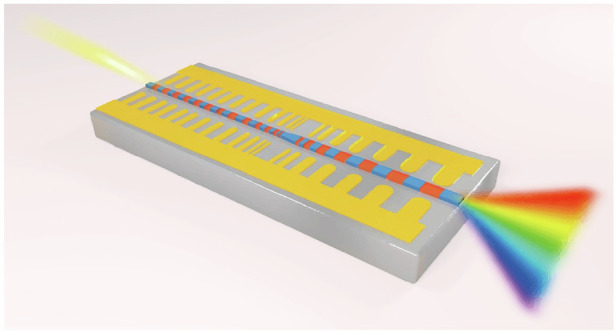

## Introduction

Supercontinuum generation (SCG) is a nonlinear process in which an ultrashort and intense optical pulse undergoes significant spectral broadening during its propagation in nonlinear optical materials. In particular, ultraviolet SCG (UV-SCG) has emerged as a transformative technology for precision spectroscopy and fundamental physics, enabling direct probing of electronic transitions through broadband spectral coverage below 400 nm^1–3^. The unique combination of ultra-short pulse duration, high peak power, and inherent phase coherence positions UV-SCG as a critical enabler for next-generation applications such as nuclear clock transitions^4^ and astronomical spectrograph calibration^5^. Particularly charming is its potential to extend frequency combs into the UV regime, bridging critical gaps in optical metrology and quantum control of atomic systems^5–7^. Supercontinuum generation in optical fibers has been extensively explored and widely applied owing to their low transmission loss and flexible dispersion engineering^8–11^. The maturation of high-nonlinearity fiber and crystal fiber fabrication technologies has greatly accelerated the development of supercontinuum, enabling fiber-based supercontinuum generation across the visible, near-infrared, and mid-infrared spectral regions. Recent advances have further extended fiber-based supercontinuum into the ultraviolet region^12–15^. These explorations of nonlinear frequency generation in the ultraviolet regime have played a pivotal role in advancing broadband ultraviolet light sources and spectroscopic sensing. In contrast to traditional SCG based on fiber and bulk nonlinear optics^16–18^, on-chip SCG has emerged as a key research focus^1,19^ to achieve highly compact supercontinuum sources with a footprint in mm^2^-scale^20^. Moreover, building upon the established foundation of on-chip photonic components such as optical filters^21^, optical modulators^22^, and optical sensing waveguides^23,24^, further developments are expected to enable monolithic integration of SCG with other specific modules for satisfy the demands of various applications^25,26^.

Although nanophotonic architectures leveraging tight mode confinement and dispersion engineering have revolutionized SCG in the infrared^1,19,27^, on-chip SCG extended to cover the UV-band still faces fundamental limitations from the intrinsic properties of conventional photonic materials. First, most integrated photonic materials exhibit prohibitive UV absorption at wavelengths below 350 nm. Second, the strong normal dispersion at the UV band for the femtosecond pump fundamentally disrupts the parametric processes through phase mismatch^2,6^. Recent attempts to circumvent these challenges through the second-order nonlinearity in aluminum nitride (AlN)^6,28,29^ and lithium niobate (LN)^7,30^ waveguides have achieved partial success by utilizing 3-wave-mixing (3WM) or cascaded 3WM. This is helpful to enhance the wavelengths in the visible and UV range, while the intrinsic material constraint remains. One should note that, AlN’s weak second-order nonlinearity with χ²~4.7 pm/V necessitates the use of extremely large pump intensities for efficient frequency conversion^6,28,29^, while LN’s UV absorption edge near 330 nm fundamentally limits spectral extension even though it has a high nonlinear coefficient (χ² ~ 25 pm/V)^31^ and also supports quasi-phase-matching (QPM) for 3WM^7,30^. Besides, in order to satisfy the phase matching condition, the UV and near-UV light of the SCG on SiN^32^ and AlN^2,6,28,29^ is often carried by the higher-order modes, which is not preferred when it is desired to integrate the SCG sources with other specific modules on a chip. Instead, the SCG is desired to operate with the fundamental mode, and one has to address the bending losses, intermode crosstalk and other challenges possibly caused by any undesired mode coupling/conversion over more than an octave-spanning bandwidth, which is especially difficult for high-birefringence material systems like LN^33^.

Here, we demonstrate an unprecedented UVC-to-mid-infrared (MIR) SCG on a chip, leveraging the exceptional transparency window and second-order nonlinearity in thin-film lithium tantalate (LT). Compared to LN, LT offers near-zero birefringence, superior UV transparency (extending to 260 nm^34^ and covering the range of 260-5500 nm), enhanced optical damage threshold, and similar χ² nonlinear coefficients ( ~ 21 pm/V)^35,36^. Previously the periodically poled lithium tantalate (PPLT) waveguide with a period of 3.25 μm for the SHG of 1064 nm CW pump was reported^37^, while it is still very challenging to develop the PPLT waveguide with submicron ferroelectric domains needed for UV phase matching, which has not been reported yet. In this paper, we overcome the longstanding fabrication barrier for achieving submicron ferroelectric domains achieved by introducing novel rounded-tip electrodes and manipulating the poling electric field. This enables simultaneous cascaded 3WM processes in a single waveguide architecture, establishing three key innovations, including: (1) A multi-period poling design spanning 0.95-10 μm domains for UV-to-MIR spectral engineering; (2) Dispersion-optimized mode confinement balancing the nonlinear efficiency from UV to MIR; (3) Wafer-scale compatibility with standard semiconductor processing techniques.

Through strategic engineering of cascaded second-harmonic generation (SHG) and sum-frequency generation (SFG) processes with optimized QPM, we demonstrate the first SCG extending to the deep-UV region at 270 nm on chip. Concurrently, MIR spectral broadening is realized through difference-frequency generation (DFG) in the same waveguide. Such nonlinear processes generates a supercontinuum exceeding three octaves on a chip for the first time, i.e., spanning continuously from <270 nm to >2400 nm, achieved even with remarkably low pump energy of 100 pJ. To our knowledge, this constitutes both the shortest-wavelength on-chip SCG reported to date and the first chip-scale SCG source encompassing the entire UVA/UVB bands (315-400 nm & 280-315 nm) and even entering the UVC regime (200-280 nm). ‌Furthermore, we present an on-chip photonic sensing system that integrates a chirped PPLT SCG source with a 2-cm-long sensing spiral waveguide, enabling the spectral analysis spanning from the UV to the IR. Here, the spiral waveguide is designed with a compact footprint of only 0.18 mm² and works well in an ultra-broadband range covering the UV–visible–infrared spectrum, by leveraging the low birefringence and positive uniaxial crystal properties of LT^35^. Experimental validation demonstrates this system’s capability for multispectral sensing of liquid/gas-phase analytes across UV, visible, and infrared domains. Notably, our UV-SCG extension to 270 nm surpasses previous chip-scale SCG limited to the UVA band, thereby unlocking the applications in atmospheric monitoring and biochemical sensing. Particularly relevant for biochemical analysis, the attained UV coverage encompasses critical n→π* electronic transitions that govern protein structural dynamics and facilitate label-free quantification of aromatic amino acids via the intrinsic absorption around 280 nm^38,39^. The present UV-SCG source additionally targets characteristic absorption bands of environmental pollutants including SO₂ ( ~ 300 nm), O₃ ( ~ 300 nm), acetone (279 nm), and α,β-unsaturated carbonyl compounds such as crotonaldehyde (290 nm)^40^, thereby establishing a versatile tool for industrial process monitoring and environmental analytics.

## Results

### Principle concept and structural design

**Fig. 1:**
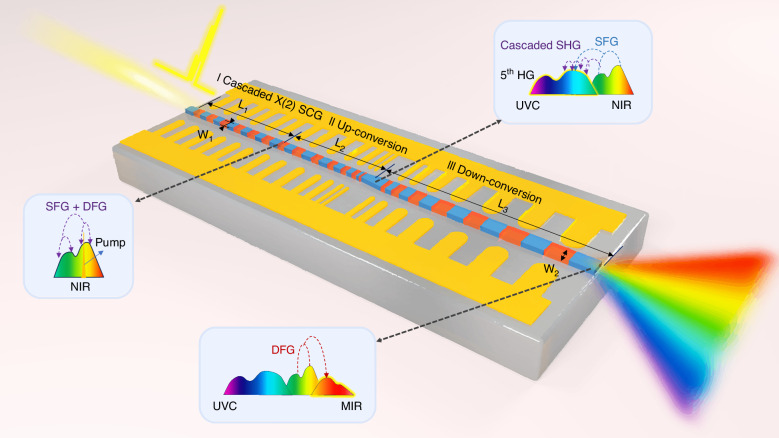
Schematic illustration of the proposed device. The structure consists of three cascaded components with varying waveguide widths and poling periods: a uniformly poled, dispersive-engineered waveguide for SCG; an up-conversion waveguide for UV and visible light generation; and a down-conversion waveguide for MIR generation

Figure 1 schematically illustrates the proposed waveguide structure for achieving UV to MIR (UV–MIR) SCG using a C-band pump laser. As shown in Fig. 1, the structure consists of three key sections (I, II, III) with varying waveguide widths and poling periods. Section I is for cascaded χ² SCG to achieve near-infrared (NIR) SCG, section II is for up-conversion to generate UV and visible light, while section III is for down-conversion to generate the MIR light. Here, in section I, efficient cascaded χ² SCG is first achieved for NIR SCG by using a uniform PPLT waveguide, whose poling period is designed for phase-matched SHG of the pump wavelengths. Meanwhile, near-zero anomalous dispersion and small group velocity mismatch (GVM) in NIR are also required. The up-conversion waveguide in section II is based on PPLT with spatially linearly decreasing poling periods, allowing the phase-matching wavelengths to vary from the visible to UV band along the propagation direction. In this section, SHG and SFG are the key mechanisms for expanding the spectra into the visible and UV ranges. In section III for down-conversion, the poling periods increase linearly to enable wide-band phase-matched DFG, further broadening the spectrum into the MIR region. Particularly, the width (W_2_) of the waveguide in section III is broadened to improve the mode confinement for the long wavelength in MIR, as analyzed in details in the supplementary material. On the other hand, for UV-visible light, such a broadened waveguide greatly reduces the power density, and thus the wavelength conversion efficiency is weakened so that the energy in the original short wavelength bands is preserved. As a consequence, the proposed waveguide structure comprising sections I/II/III facilitates the SCG from UV to MIR. Since the waveguide features a slowly varying structure with a fixed propagation direction, and its operation relies on the TE₀ mode only, the birefringence of LT does not limit the bandwidth of the generated supercontinuum.

**Fig. 2:**
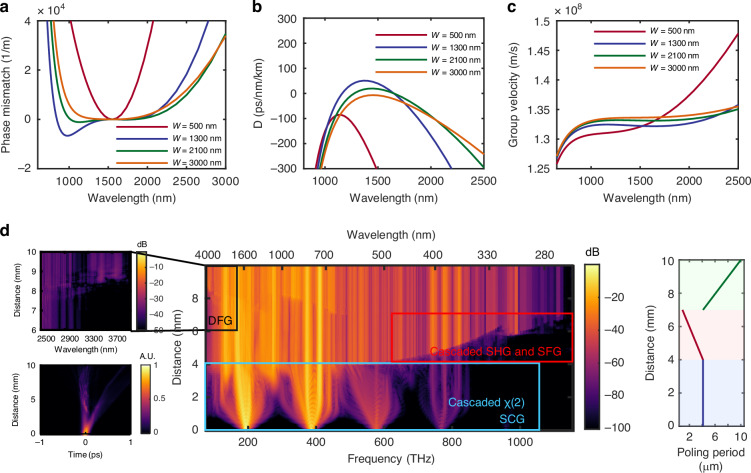
The simulation of the proposed device. **a**–**c** The phase mismatch, dispersion and group velocity of LT-on-insulator waveguides at different widths of W = 500, 1300, 2100 and 3000 nm, respectively. **d** On left bottom is the time evolution on the PPLT waveguides, and in the center is the frequency evolution on the PPLT waveguides. On the right is the poling period as a function of the propagation distance of this structure. In all the simulation it is slab waveguide with Height=600 nm and etch depth=300 nm

Here, a LT-on-insulator (LTOI) wafer with a 600-nm-thick LT core layer and a 4.7-μm-thick buried-oxide layer is used, and the etch depth is chosen to be 300 nm. Figure 2a–c shows calculation results of the phase mismatch, dispersion, and group velocity of LTOI photonic waveguides with different widths of W = 500 nm, 1300 nm, 2100 nm, and 3000 nm, respectively. As observed, the waveguides exhibit anomalous dispersion at the pump wavelength of 1550 nm when the width is chosen as 1300 nm or 2100 nm. In particular, the 1300-nm-wide waveguide exhibits the smallest GVM of 1.027×10^-10 ^s/m between the pump and SHG lights, resulting in enhanced effective self-phase modulation (SPM) due to cascaded second-order nonlinearity^41^. Moreover, smaller mode area contributes to higher effective nonlinear coefficients. As a consequence, we choose the width W_1_ as 1300 nm for the uniform PPLT waveguide in section I in our design. Here the etching depth was not specifically optimized, as they are designed to be compatible with our fabrication process. Dispersion calculations for different etching depths are included in supplementary material.

Figure 2d shows the simulated time- and frequency-domain evolution along the PPLT waveguide with the following parameters: W_1_ = 1300 nm, W_2_ = 3000 nm, L_1_ = 4 mm, L_2_ = 3 mm and L_3_ = 4 mm. In this simulation, we use100-fs pulse pump at 1550 nm. Considering both the final bandwidth performance of the SC source and the cost of the pump femtosecond laser, we choose a on-chip pulse energy of 100 pJ, which corresponds to an on-chip average power of 10 mW (see more details in supplementary material). Initially, the poling period is set to be 4.13 μm. The incident pulses undergo strong temporal compression, followed by soliton fission occurring at the propagation distance of z = ~2 mm, which leads to significant spectral broadening. SHG and THG are also observed around the wavelengths of 775 nm and 515 nm, respectively. At the propagation distance of z = 4 mm, the energy in the visible, especially in the UV range is still weak, mainly due to strong SPM phase mismatch occurring at these wavelengths. After propagating through the chirped PPLT waveguide for up-conversion in the distance from z = 4 mm to z = 7 mm, where the poling period decreases from 4.13 μm to 0.95 μm, the χ² phase-matching condition is sequentially satisfied for light in the wavelength range from NIR to the visible and eventually to UV. As shown in Fig. 2d, the short-wavelength edge even reaches 270 nm, covering the entire UVA/UVB bands and extending to the UVC band, indicating that the on-chip fifth harmonic generation (5th HG) of the C-band pump is achieved. Finally, for the down-conversion PPLT waveguide of section III (i.e., 7 mm < z < 10 mm), the poling period increases from 4.13 μm to 10 μm to satisfy the QPM condition for DFG, resulting in further spectral expansion and enhancement in the MIR region. As a result, the achieved SCG spectrum spans from 270 nm (UVC) to > 4000 nm (MIR).

### Fabrication and measurement

**Fig. 3:**
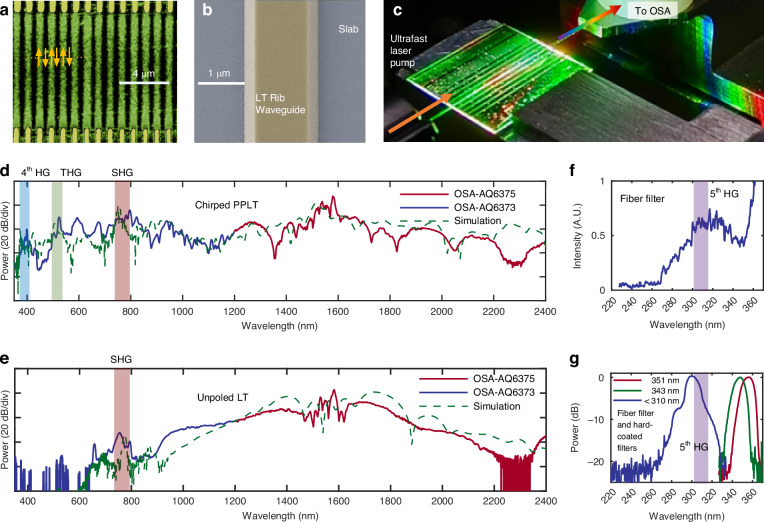
UVC to MIR SCG. **a** False-color PFM image of the PPLT with a poling period of 0.95 µm. **b** SEM image of fabricated waveguide. **c** Photograph of a glowing PPLT chip under test. **d**, **e** Measured and simulated SCG output spectrum from a chirped PPLT waveguide and unpoled waveguide. SHG, third-harmonic generation (THG), and fourth-harmonic generation (4th HG), are labeled in distinct colors. The green and pink traces represent measurements using optical spectrum analyzers AQ6373 (350–1200 nm) and AQ6375 (1200–2400 nm) from YOKOGAWA, respectively. **f** Measured UV SCG output from the chirped PPLT waveguide after the rotary fiber filter. **g** Measured SCG spectra after additional filtering by a 351-nm bandpass filter, a 343-nm bandpass filter, and a 310-nm short-pass filter. The corresponding fifth harmonic generation (5th HG) wavelengths of the 1550-nm pump are also indicated. All experiments were conducted using 1550-nm pump pulses with 100 MHz repetition rate and 100 fs pulse duration

The designed PPLT waveguide was fabricated using regular lithography and etching processes without any special requirement. Here our design introduces a gradual transition in the poling period from 0.95 µm to 10 µm, which presents some significant challenge for ferroelectric domains, particularly for submicron ferroelectric domains, which is a long-standing difficulty for poling techniques. To improve the poling quality, we employ three strategies. First, experimental observations revealed that, for a fixed electrode duty cycle, smaller periods yield wider inverted domains especially for submicron periods. To compensate for this, the duty cycle of the poling electrodes is increased from 10% to 50% for the case when the poling period increases from 0.95 µm to 4 µm, and maintained at 50% when the period is larger than 4 µm. Second, since the waveguide comprises three cascaded sections with different poling periods, the electrodes are divided into three independently patterned segments. This allows the fine-tuning of poling parameters for each section, thereby improving the poling quality separately. Finally, due to the non-uniformity of nucleation across the wafer, poling defects may occur in certain regions. To mitigate this, a high-voltage pre-poling pulse (700 V) opposite in polarity to the main poling voltage is applied prior to the formal poling process, which is found to greatly reduce poling defects. Figure 3a shows the false-color Piezoresponse Force Microscopy (PFM) image of the fabricated PPLT, demonstrating that the poling is uniform and exhibits an appropriate duty cycle, even at a submicron poling period of 0.95 μm. After poling, waveguides are defined using the electron-beam lithography (EBL) and etched using Ar⁺ inductively coupled plasma (ICP) etching of LT. Compared with wet etching, Ar⁺ dry etching often exhibits a much weaker dependence on the ferroelectric domain orientation^31^. The Scanning Electron Microscope (SEM) image of the fabricated waveguides with a poling period of approximately 1 µm is shown in Fig. 3b. As observed, the LT waveguide has high etching quality and uniform core-width, and there are no periodic surface features induced by domain polarization.

In our experiment, we use a 100-fs pulse pump at 1550 nm with a pulse energy of 100 pJ on the chip, and the in-waveguide peak power is 1000 W. It should be noticed that LT exhibits a significantly higher optical damage threshold (1064 nm, 240 MW/cm²) compared to LN (1064 nm, 120 MW/cm²). Due to the insufficient power of the femtosecond laser available in our lab, we were unable to experimentally test the damage threshold for the PPLT waveguide. On the other hand, we found that the LT waveguide could safely handle the continuous-wave (CW) light at 1550 nm without damage even when the on-chip power is as high as 30 dBm (1 W). The pump pulses are butt-coupled into the waveguide using a lensed fiber, resulting in a coupling loss of approximately 10 dB for the pulse pump. We replaced the original output fiber (PM1550, approximately 1 m in length) of the femtosecond laser with a lensed fiber of the same type and length to eliminate the influence of the coupling fiber on the pulse parameters. The propagation loss of the fabricated 1.3-μm-wide LT photonic waveguides were measured with the cut-back method to be ~0.5 dB/cm at 1550 nm, ~0.6 dB/cm and 1064 nm and ~0.8 dB/cm at 400 nm. The output light was collected using a polarization-maintaining (PM) lensed fiber optimized for 1550 nm and directed to two optical spectrum analyzers including AQ6373 YOKOGAWA (350–1200 nm) and AQ6375 YOKOGAWA (1200–2400 nm). The resolution of spectrometer is set to be 2 nm. Figure 3c shows the photograph of a glowing PPLT chip under test, and Fig. 3d displays the measured and simulated spectra output from the chirped PPLT waveguides, covering a broad range from 350 nm to 2400 nm. The results show that the chirped PPLT waveguide enables continuous supercontinuum spectrum with a spectral bandwidth extending from below 350 nm to beyond 2400 nm. The measured total output power is -0.88 dBm. The measured conversion efficiency from the near-infrared pump to the ultraviolet–visible region of 350–760 nm is 19.3%. In the simulation, we modified our model by introducing a small phase mismatch Δβ = 7.3 (mm^-1^) in section I by considering the mismatch arising from the fabrication imperfection in the PPLT waveguide, such as variations in the film thickness, the waveguide width, and the etching depth. A detailed discussion of the impact of this phase mismatch on the supercontinuum generation results is provided in the Supplementary Materials. After incorporating this correction, the simulation results show good agreement with the experimental one. Note that such fabrication-induced mismatches can be effectively compensated through adaptive poling techniques^42^. For comparison, we also measured and simulated the spectrum generated with an unpoled LT waveguide at the same pump condition, as shown in Fig. 3e. It can be seen that the produced continuous spectrum ranges from ~510 nm to 2230 nm, which is considerably narrower and more irregular compared to the present SCG from the chirped PPLT waveguide. Furthermore, Fig. 3e also shows that the third-harmonic generation (THG) and fourth-harmonic generation signals are below the detection limit (i.e., ~ -70 dBm/nm) of the commercial spectrometer. Basically speaking, the lack of phase matching causes low conversion efficiency in the unpoled LT waveguide. In contrast, the chirped PPLT waveguides exhibit a much broader and flatter supercontinuum spectrum than the unpoled counterparts, particularly in the visible, ultraviolet, and MIR regions. Our laboratory currently lacks an optical spectrometer for the wavelength range beyond 2400 nm; therefore, the chip performance in this spectral region remains uncharacterized. In Supplementary Materials, we present the numerical simulation results to compare the complete waveguide comprising Sections I–III and a truncated waveguide only with Sections I–II. It is shown that the complete waveguide exhibits a significant enhancement relative to the truncated waveguide within the 3300–3800 nm wavelength range, confirming that Section III plays a key role for facilitating the down-conversion process to achieve spectral broadening in the long wavelength-band. And MIR via femtosecond-laser-driven DFG has already been experimentally demonstrated and theoretically analyzed in PPLN crystals^43^. The measured SNR at 750 nm is ≥37.6 dB. One should note that the SNR measurement is limited by the PD sensitivity rather than the SC waveguide itself. A higher-power pump source is required for measuring the true SNR of the chirped PPLT, but it is currently unavailable in our lab. Nevertheless, the SNR higher than 37.6 dB is usually sufficient for most biological and chemical sensing applications. Previous studies on PPLN have reported that carrier–envelope offset frequency (f_ceo_) detection, also confirming the performance excellence of SNR in the spectral range from ultraviolet to visible^30^. And it should be noticed that the noise-generating processes associated with such massive spectral broadening involving both χ ^(2)^ and χ^(3)^ nonlinearities remain an important topic for future investigation.

For UV spectral measurements, we used a UV single-mode fiber (SM300-SC) from FIBERCORE for output collection. A primary challenge is the crosstalk from longer wavelengths because the stray light in the visible and NIR ranges may be mistakenly detected as UV if not blocked. However, currently there is no bandpass filter available to transmit the UV light but block visible/NIR light. To address this, we wrapped the collection fiber around a cylinder with a diameter of 2 cm to induce a high bending loss for wavelengths longer than the fiber’s cutoff (430 nm), effectively functioning as a short-pass filter. Such a fiber filter has a transmission dropped to <0.001 at the wavelength of 500 nm in experiment, which is beyond the measurement limit of a high-performance CCD (ILX511), achieving high extinction ratios comparable to or better than many commercial spectral filters. The filtered spectrum is then analyzed using an Ocean Optics USB 2000+ optical spectrum analyzer. Figure 3f presents the measured output spectrum of the chirped PPLT waveguides after the fiber filter, demonstrating that continuous SCG extending beyond 270 nm is achieved, thus covering both the UVB and UVA bands. To further validate these results, we measured the spectrum after the fiber filter by introducing additional three separate optical filters, i.e., a 351 nm bandpass filter (FBH351-10, FWHM 10 nm, Thorlabs), a 343 nm bandpass filter (FBH343-10, FWHM 10 nm, Thorlabs), and a 310 nm short-pass filter (XUV0310, Asahi Spectra). These measurements confirm the presence of spectral components near 351 nm, 343 nm, and 300 nm, as shown in Fig. 3g, providing strong evidence that the supercontinuum (SC) spectrum extends to the fifth harmonic generation ( ~ 310 nm). Note that the shortest wavelength detected after filtering is approximately 270 nm, which aligns well with our simulation results, while the UV bandwidth limitation is likely due to the LT absorption which increases rapidly for wavelengths below 280 nm.

### UV to infrared (IR) absorption spectroscopy

The supercontinuum is a promising broadband light source for optical absorption spectroscopy. Conventionally, a fiber-based supercontinuum source is often used in the spectroscopy system together with the cuvettes for sample absorption and a spectrometer for detection^44,45^. Since it’s important for the sensing waveguide to be compact and low-loss across a wide range of wavelengths, we introduce a compact and wide-band waveguide spiral on the chip to work as the sensing element. Compared to LN, whose anisotropy is as high as -0.07^35^, LT offers more than 10-fold reduced birefringence ( ~ 0.004)^35^, making it a more suitable platform for ultra-low-loss, densely integrated spiral waveguides for enhanced light–matter interaction. In the supplementary material, we compare the effective refractive indices of bending LN and LT photonic waveguides. It can be seen that the TE_0_ mode in the LN photonic waveguide exhibits notable mode hybridizations with the TM_1_ mode at around 900 nm and with the TM_0_ mode at 1350 nm when the bending radius is 100 µm. In contrast, there is no mode hybridization found for the LT photonic waveguide even at a small bending radius of 60 µm.

**Fig. 4:**
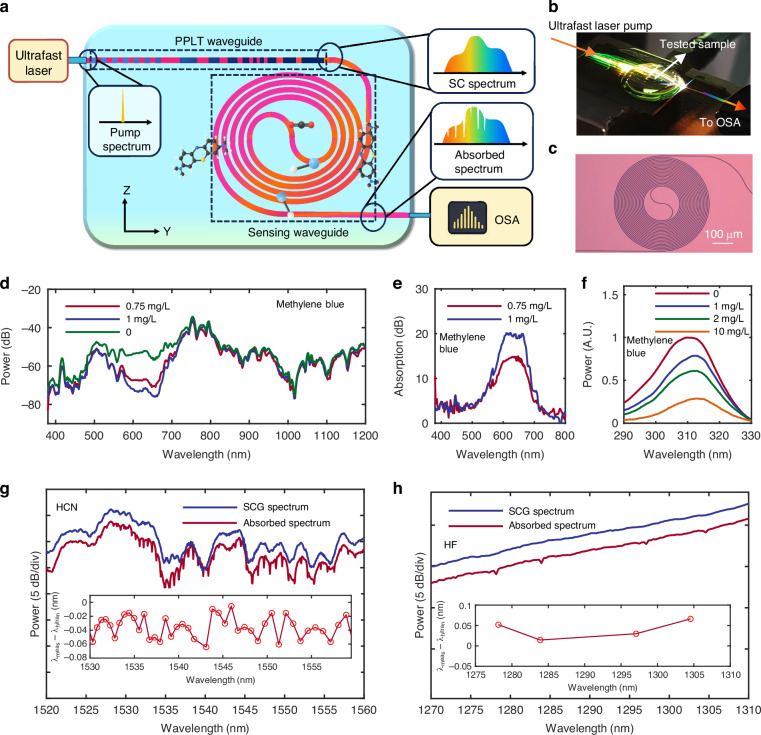
UV-to-IR spectral sensing using the present chirp PPLT supercontinuum light source. **a** Experimental setup for on-chip spectral sensing. **b** Photograph of the on-chip UV-to-IR spectral sensing chip under test. The scattered light from the on-chip supercontinuum sources is clearly visible, and the droplet on top of the chip is the test sample. **c** Microscope images of the fabricated waveguide spiral for sensing. **d** Measured UV-visible transmission spectra of air and aqueous methylene blue solutions with the concentrations of 0.75 mg/L and 1 mg/L. **e** UV-visible absorbance spectra of methylene blue solutions at different concentrations, obtained by normalizing each sample spectrum to the spectrum measured with no solution on the waveguide spiral. **f** Measured UV transmission spectra of cuvettes containing methylene blue solutions at concentrations of 0 (empty), 1, 2, and 10 mg/L. **g** Measured transmitted IR spectrum with or without the HCN gas cells, respectively. The insets display the wavelength differences of the measured absorption peaks of HCN relative to the HITRAN database.^48^ **h** Measured transmitted IR spectrum with or without the HF gas cells, respectively. The insets display the wavelength differences of the measured absorption peaks of HF relative to the HITRAN database^48^

In this study, we present an on-chip UV-to-IR spectral sensing platform that integrates a chirped poled PPLT supercontinuum source with a sensing waveguide on the same chip. The platform enables sensing and spectroscopy of various liquid and gas samples across the UV, visible, and IR wavelength-bands. Figure 4a illustrates the experimental setup for the on-chip UV-to-IR spectral sensing. The pump pulse is butt-coupled into the PPLT waveguide, generating a broad-band SCG light. The following light-matter interaction occurs in the 2-cm long waveguide spiral, while the transmitted spectrum is measured using an optical spectrum analyzer, with data processing performed by a computer. Figure 4b shows a photograph of the on-chip UV-to-IR spectral sensing chip under test, while Fig. 4c presents an image of the spiral sensing waveguide on LTOI.

First, we investigate the absorption spectroscopy of methylene blue (C₁₆H₁₈N₃ClS) aqueous solutions with different concentrations. As it is well known, methylene blue is widely used for staining tissues and bodily fluids before or during surgery and diagnostic procedures, as well as serving as an antiseptic, an internal wound-healing agent, and a dye in microscopic analysis^46,47^. Thus, measuring methylene blue concentration is highly relevant in the applications such as activated carbon performance evaluation and concentration analysis of polymeric amino acid compounds. As shown in Fig. 4d, we measured the transmitted spectra in the UV-visible-IR range for air, and methylene blue solutions with the concentration of 0.75 and 1 mg/L, respectively. It can be seen that, when the concentration increases, the absorption increases as well. Figure 4e presents the normalized absorption spectra with respective to the measured spectrum for the case without samples. A strong absorption peak appears around 660 nm, corresponding to a commonly used spectral signature for methylene blue detection. We also conducted UV-absorption measurements for the light from the PPLT waveguide, passed through a 2 cm-long cuvette containing either methylene blue solution or air, followed by a 310 nm short-pass filter and, finally, an optical spectrum analyzer. The results are shown in Fig. 4f. As it can be seen, the absorption increases as the concentration of methylene blue increases. As a consequence, our chirped poled PPLT supercontinuum source is capable of UV to visible liquid detection with both supercontinuum source and sensing spiral on chip.

Moreover, we also performed IR absorption spectroscopy for H_13_C_14_N (25 Torr, 55 mm cell path length, Technicasa) and HF (50 torr, 27 mm cell path length, Wavelength references) using our PPLT supercontinuum source. The light from the PPLT waveguide in the spectroscopy module is fiber-coupled into the gas cells and the transmitted spectrum are sent to an OSA for analysis. Figure 4g, h shows the measured transmitted IR spectra with and without the HCN and HF gas cells, respectively. The insets display the wavelength differences of the measured absorption peaks of HCN and HF relative to the HITRAN database^48^. It shows that the mean wavelength differences are −0.0329 nm and 0.045 nm, respectively, and the corresponding variances are 0.00040 and 0.00053. During the experiment, coupling between the optical fiber and the chip exhibited certain instability, which was a major source of error in gas absorption measurements. Currently, the measurement accuracy is primarily limited by the precision of the commercial OSA used (MS9740B, Anritsu). In the future, the implementation of an on-chip spectrometer based on high-Q micro-resonators^49^ on the same chip could significantly improve measurement accuracy and reduce coupling instability.

## Discussion

**Fig. 5:**
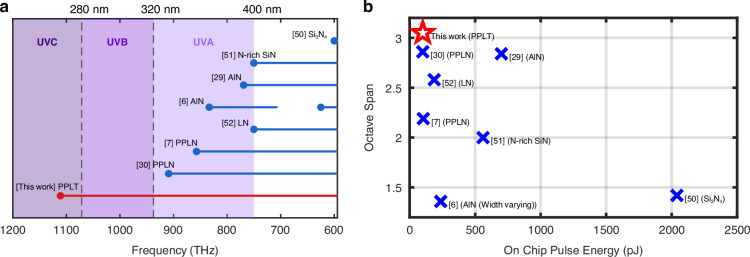
Performance comparison of supercontinuum light sources reaching or approaching the ultraviolet spectral region. **a** A comparison between the ultraviolet wavelength range covered in this work and with previous demonstrations. **b** Comparison of the pulse energy requirement and achieved octave span by this work with previous demonstrations

Figure 5a presents a comparison between the UV light achieved in this work and those reported in previous demonstrations of on-chip SCG reaching or approaching the UV region^6,7,29,30,50–52^. Our results surpass the previous UV bandwidth limitations of on-chip SCG by extending the supercontinuum emissions into the UVC band. To the best of our knowledge, this is the first on-chip SCG that reaches the UVC region. Moreover, it is not only the first to cover the entire UVA band but also the first to span the entire UVB band. Additionally, the generated spectrum is continuous from the UVC to the MIR region, highlighting its strong potential for ultra-broadband frequency metrology.

In contrast, for SiN photonic waveguides^50,51^, the absence of second-order nonlinear effects restricts their performance in the UV region, and none of the reported works have achieved UV-band SCG. The inability to achieve quasi-phase matching in AlN and the significant UV absorption in LN are the main limitations for these platforms, resulting in all reported SCG in AlN and LN to be limited to the wavelength-band above 330 nm^6,7,29,30,52^. Figure 5b compares the on chip pulse energy requirements and octave-spanning capabilities of our work with those previous demonstrations, showing that the present device is the first UV-IR SCG that achieves a span exceeding three octaves on chip. Furthermore, the present results are achieved even operating with a low pulse energy of just 100 pJ, which is owing to the chirped QPM design introduced in this paper. In summary, our work breaks the existing UV bandwidth limitations of on-chip SCG by covering the full UVA and UVB bands, extending into the UVC band and achieving a continuous spectrum from UVC to MIR.

In conclusion, we have demonstrated on-chip UVC to MIR SCG using chirped PPLT. We have overcome the difficulty of achieving submicron ferroelectric domains and realized 0.95 to 10 um poling period in thin film LT waveguides by introducing novel rounded-tip electrodes and manipulating the poling electric field. To the best of our knowledge, we achieve the smallest poling period realized in LT photonic waveguides. By cascading SHG and SFG under optimized QPM conditions, we demonstrate the supercontinuum emission extending to 270 nm in the UVC band - the shortest wavelength reported for on-chip SCG. Concurrently, enhanced mid-infrared SCG is achieved via DFG within the same waveguide. The resulting supercontinuum emission spans continuously from <270 nm to >2400 nm, encompassing more than three octaves, achieved with just 100 pJ pump energy. Compared with visible–UV supercontinuum generation schemes based on modal phase matching^6,29^, our approach employs a chirped poling design to achieve quasi-phase matching for the fundamental mode, thereby concentrating the generated spectral energy more effectively in the fundamental mode, as further discussed in the Supplementary Materials. We have also demonstrated the application of this supercontinuum source for sensing and spectroscopy of various analytes, including liquids and gases, across the ultraviolet, visible, and infrared wavelength-bands. To the best of our knowledge, this work represents the first on-chip SCG fully covering the UVA/UVB bands while extending into the UVC band with the shortest-wavelength to date. In addition to integrated photonic platforms, significant progress has also been made in UV supercontinuum generation based on optical fibers. For example, hollow-core fibers have been demonstrated to produce a broadband supercontinuum spanning from 200 nm to 1.7 μm with a pulse energy of approximately 23 μJ^53^, while solid-core ZBLAN photonic crystal fibers can generate spectra covering 200–2500 nm with launched pulse energy around 1000 pJ^13^. Compared with on-chip supercontinuum generation, these fiber-based approaches typically require higher pump powers and longer propagation lengths but are capable of extending the supercontinuum further into the shorter-wavelength UV region. Additionally, solid-state materials^54^, gases^55^ and liquids^56^ also hold great potential for UV supercontinuum generation. Heterogeneous integration of these promising media with on-chip waveguides such as PPLT may further enhance the performance of on-chip supercontinuum sources. The proposed supercontinuum generated in the chirped PPLT waveguide shows great potential for applications in dual-comb spectroscopy^18,57,58^. For example, the chirped PPLT waveguide is useful as a wavelength conversion platform^7,59,60^ for optical frequency combs, to enable the transfer of dual-comb signals—typically operating in the near-infrared—into UV, visible, or MIR spectral regions. In addition, the PPLT waveguide can provide an f–2 f self-referencing signal for frequency comb stabilization, facilitating f_ceo_ locking^26^. One should note that high-efficiency edge couplers are necessary for the real applications to avoid high fiber-chip coupling losses^61^. Our work has shown great potential of LTOI photonics for full-spectrum nonlinear photonics, opening new possibilities for on-chip UV light sources, and may find promising applications in on-chip spectroscopic sensing^27^, gas monitoring^59^ or astronomical spectrograph calibration^5^.

## Materials and methods

### Device fabrication

Devices were fabricated on a 600-nm x-cut LT on a 4.7-µm-thick silica buried oxide layer with a 525-µm-thick silicon substrate. First, the poling electrodes were patterned by a step of electron-beam lithography (EBL), a layer of 10/100-nm Ti/Au electrodes were deposited by the steps of electron-beam evaporation and lift-off processes. The duty ratio of poling the electrodes increased from 10% to 50% as the poling period increased from 0.95 µm to 4 µm and is kept to be 50% when the poling period is larger than 4 µm. Second, a 700 V voltage pre-poling pulse opposite in polarity to the main poling voltage was applied. Third, we performed the periodic domain inversion by applying several 280 V, 6-ms-long pulses at room temperature with the sample submerged in oil. After periodically poling, the electrodes are removed in potassium iodide solution. Then, the LT photonic waveguides were patterned by a EBL, and finally the pattern was transferred into the LT layer by Ar^+^ plasma dry etch, in which way the etching depth was 300 nm and the etched waveguide ridge had a sidewall angle α ≈ 60°.

### Numerical simulations

The phase-matching wavelengths for 3WM in a PPLT waveguide with a period Λ can be calculated using the standard phase-matching condition^43^

1 $$m\frac{2\pi }{\Lambda }=\,\beta \left(\omega \right)-{\beta }_{1}\left({\omega }_{1}\right)\omega +2{\beta }_{1}\left({\omega }_{1}\right){\omega }_{1}-2\beta \left({\omega }_{1}\right)-\gamma {P}_{s}\left(\omega > {\omega }_{1}\right)(\mathrm{SFG})$$

2 $$m\frac{2\pi }{\Lambda }=-\beta \left(\omega \right)+{\beta }_{1}\left({\omega }_{1}\right)\omega \left(\omega < {\omega }_{1}\right)({\rm{DFG}})$$

3 $$m\frac{2\pi }{\Lambda }=2\beta \left(\omega /2\right)-\beta \left(\omega \right)({\rm{Cascaded\; SHG}})$$

Here, *ω* and *ω₁* denote the phase-matching frequency and the pump frequency, respectively, *β* is the propagation constant; *P*_*s*_ represents the peak power of the first ejected soliton; *γ* is the nonlinear coefficient; *β₁* is the first-order dispersion coefficient; and *m* = 1, 2, 3… corresponds to the Fourier order of the nonlinear coupling coefficient. QPM is most effectively achieved using a first-order interaction (*m* = 1), where the nonlinear coupling coefficient attains its maximum value. In this system, SFG and cascaded SHG contribute to spectral broadening in the visible and UV regions, while DFG facilitates spectral expansion into the MIR region.

Single nonlinear envelope equation considering the χ^2^ and χ^3^ effects to model ultra-broadband nonlinear SCG in the PPLT waveguide^43,62–65^, i.e.,

4 $$\left[\frac{\partial }{\partial z}-i\mathop{\sum }\limits_{n{\ge }{2}}\frac{{\beta }_{n}}{n!}{\left(i\frac{\partial }{\partial t}\right)}^{n}+\frac{\alpha }{2}\right]E(z,t)=i\frac{{\omega }_{1}}{2{n}_{0}c}\left[1+i\left({\left.\frac{1}{{\omega }_{1}}-\frac{\partial {\mathrm{ln}}(n(\omega ))}{\partial \omega }\right|}_{\omega ={\omega }_{1}}\right)\frac{\partial }{\partial \tau }\right]\left[{\chi }^{(2)}(z){E}^{2}+{\chi }^{(3)}{E}^{3}\right]$$

where ${\epsilon }_{0}$ is the permittivity in free space, n0 is the effective index, ${\beta }_{n}$ is the n-th order dispersion coefficient, α is the propagation loss, and $\tau =t-{\beta }_{1}{\rm{z}}$ is the local time in the moving frame and E is the electric field, ${\chi }^{\left(2\right)}{E}^{2}{\rm{and}}{\chi }^{(3)}{E}^{3}$ are the second and third order of nonlinear polarizations, respectively. Equation (3) is handled numerically via the split-step Fourier method with the nonlinear step solved in the time domain and the dispersion step in the frequency domain. The sign of${\chi }^{\left(2\right)}$ varies along the propagation direction due to the domain inversion, so ${\chi }^{\left(2\right)}\left(z\right)$ is explicitly treated as a function of the propagation distance and updated accordingly during the simulation. We use Lumerical MODE to calculate the effective refractive index and propagation constant of the TE₀ mode in the LT photonic waveguide. The simulation model incorporated the birefringent property of LT, fully accounting for the influence of birefringence on the refractive index.

## Supplementary information

Supplementary Materials for Ultraviolet-C to Mid-infrared Supercontinuum Generation in Periodically Poled Lithium Tantalate Waveguides

## Data Availability

The data that support the findings of this study are available from the corresponding author upon reasonable request.

## References

[1] Brès, C. S. et al. Supercontinuum in integrated photonics: generation, applications, challenges, and perspectives. *Nanophotonics* **12**, 1199–1244 (2023). 36969949 10.1515/nanoph-2022-0749 PMC10031268

[2] Li, N. X. et al. Aluminium nitride integrated photonics: a review. *Nanophotonics* **10**, 2347–2387 (2021).

[3] Xu, B. X. et al. Near-ultraviolet photon-counting dual-comb spectroscopy. *Nature* **627**, 289–294 (2024). 38448594 10.1038/s41586-024-07094-9 PMC10937374

[4] Zhang, C. K. et al. Frequency ratio of the ^229m^Th nuclear isomeric transition and the ^87^Sr atomic clock. *Nature* **633**, 63–70 (2024). 39232152 10.1038/s41586-024-07839-6

[5] Cheng, Y. S. et al. Continuous ultraviolet to blue-green astrocomb. *Nat. Commun.* **15**, 1466 (2024). 38368423 10.1038/s41467-024-45924-6 PMC10874390

[6] Liu, X. W. et al. Beyond 100 THz-spanning ultraviolet frequency combs in a non- centrosymmetric crystalline waveguide. *Nat. Commun.* **10**, 2971 (2019). 31278261 10.1038/s41467-019-11034-x PMC6611800

[7] Ludwig, M. et al. Ultraviolet astronomical spectrograph calibration with laser frequency combs from nanophotonic lithium niobate waveguides. *Nat. Commun.* **15**, 7614 (2024). 39223131 10.1038/s41467-024-51560-x PMC11369296

[8] Dudley, J. M., Genty, G. & Coen, S. Supercontinuum generation in photonic crystal fiber. *Rev. Mod. Phys.* **78**, 1135–1184 (2006).

[9] Herrmann, J. et al. Experimental evidence for supercontinuum generation by fission of higher-order solitons in photonic fibers. *Phys. Rev. Lett.* **88**, 173901 (2002). 12005754 10.1103/PhysRevLett.88.173901

[10] Husakou, A. V. & Herrmann, J. Supercontinuum generation of higher-order solitons by fission in photonic crystal fibers. *Phys. Rev. Lett.* **87**, 203901 (2001). 11690475 10.1103/PhysRevLett.87.203901

[11] Husakou, A. V. & Herrmann, J. Supercontinuum generation, four-wave mixing, and fission of higher-order solitons in photonic-crystal fibers. *J. Optical Soc. Am. B* **19**, 2171–2182 (2002).

[12] Russell, P. S. J. et al. Hollow-core photonic crystal fibres for gas-based nonlinear optics. *Nat. Photonics* **8**, 278–286 (2014).

[13] Jiang, X. et al. Deep-ultraviolet to mid-infrared supercontinuum generated in solid-core ZBLAN photonic crystal fibre. *Nat. Photonics* **9**, 133–139 (2015).

[14] Ermolov, A. et al. Supercontinuum generation in the vacuum ultraviolet through dispersive-wave and soliton-plasma interaction in a noble-gas-filled hollow-core photonic crystal fiber. *Phys. Rev. A* **92**, 033821 (2015).

[15] Cassataro, M. et al. Generation of broadband mid-IR and UV light in gas-filled single-ring hollow-core PCF. *Opt. Express* **25**, 7637–7644 (2017). 28380883 10.1364/OE.25.007637

[16] Ren, H. N. et al. Low-loss silicon core fibre platform for mid-infrared nonlinear photonics. *Light Sci. Appl.* **8**, 105 (2019). 31798844 10.1038/s41377-019-0217-z PMC6872570

[17] Wang, G. et al. Stable high-peak-power fiber supercontinuum generation for adaptive femtosecond biophotonics. *Adv. Photonics Nexus* **3**, 046012 (2024).

[18] Diddams, S. A., Vahala, K. & Udem, T. Optical frequency combs: coherently uniting the electromagnetic spectrum. *Science* **369**, eaay3676 (2020). 32675346 10.1126/science.aay3676

[19] Fang, Y. X. et al. Recent progress of supercontinuum generation in nanophotonic waveguides. *Laser Photonics Rev.* **17**, 2200205 (2023).

[20] Borghi, M. et al. Nonlinear silicon photonics. *J. Opt.* **19**, 093002 (2017).

[21] Liu, D. J. et al. High-performance silicon photonic filter using subwavelength-structure multimode waveguide gratings. *Laser Photonics Rev.* **17**, 2300485 (2023).

[22] Zhang, M. et al. Integrated lithium niobate electro-optic modulators: when performance meets scalability. *Optica* **8**, 652–667 (2021).

[23] Amarloo, H. & Safavi-Naeini, S. Enhanced on-chip terahertz vibrational absorption spectroscopy using evanescent fields in silicon waveguide structures. *Opt. Express* **29**, 17343–17352 (2021). 34154279 10.1364/OE.424414

[24] Salaj, J. et al. Suspended nanophotonic waveguide for isotope-specific CO_2_ detection. *Optica* **11**, 1654–1662 (2024).

[25] Ji, X. C. et al. Millimeter-scale chip–based supercontinuum generation for optical coherence tomography. *Sci. Adv.* **7**, eabg8869 (2021). 34533990 10.1126/sciadv.abg8869 PMC8448444

[26] Okawachi, Y. et al. Chip-based self-referencing using integrated lithium niobate waveguides. *Optica* **7**, 702–707 (2020).

[27] Lafforgue, C. et al. Supercontinuum generation in silicon photonics platforms. *Photonics Res.* **10**, A43–A56 (2022).

[28] Hickstein, D. D. et al. Ultrabroadband supercontinuum generation and frequency-comb stabilization using on-chip waveguides with both cubic and quadratic nonlinearities. *Phys. Rev. Appl.* **8**, 014025 (2017).

[29] Lu, J. J. et al. Ultraviolet to mid-infrared supercontinuum generation in single-crystalline aluminum nitride waveguides. *Opt. Lett.* **45**, 4499 (2020). 32796993 10.1364/OL.398257

[30] Wu, T. H. et al. Visible-to-ultraviolet frequency comb generation in lithium niobate nanophotonic waveguides. *Nat. Photonics* **18**, 218–223 (2024).

[31] Wang, C. et al. Ultrahigh-efficiency wavelength conversion in nanophotonic periodically poled lithium niobate waveguides. *Optica* **5**, 1438–1441 (2018).

[32] Hickstein, D. D. et al. Quasi-phase-matched supercontinuum generation in photonic waveguides. *Phys. Rev. Lett.* **120**, 053903 (2018). 29481199 10.1103/PhysRevLett.120.053903

[33] Pan, B. C. et al. Compact racetrack resonator on LiNbO_3_. *J. Lightwave Technol.* **39**, 1770–1776 (2021).

[34] Ravi, G. et al. Effect of niobium substitution in stoichiometric lithium tantalate (SLT) single crystals. *J. Cryst. Growth* **250**, 146–151 (2003).

[35] Wang, C. L. et al. Lithium tantalate photonic integrated circuits for volume manufacturing. *Nature* **629**, 784–790 (2024). 38720075 10.1038/s41586-024-07369-1 PMC11111398

[36] Wang, H. H. et al. Optical switch with an ultralow DC drift based on thin-film lithium tantalate. *Opt. Lett.* **49**, 5019–5022 (2024). 39270219 10.1364/OL.531263

[37] Chen, H. W. et al. Continuous-wave second-harmonic generation of green light in periodically poled thin-film lithium tantalate. *Opt. Lett.* **50**, 1125–1127 (2025). 39951743 10.1364/OL.547762

[38] Newberry, R. W. & Raines, R. T. The *n*→*π** interaction. *Acc. Chem. Res.* **50**, 1838–1846 (2017). 28735540 10.1021/acs.accounts.7b00121 PMC5559721

[39] Bartlett, G. J. et al. *n*→*π** interactions in proteins. *Nat. Chem. Biol.* **6**, 615–620 (2010). 20622857 10.1038/nchembio.406 PMC2921280

[40] Esfandiary, R. et al. Temperature dependent 2^nd^ derivative absorbance spectroscopy of aromatic amino acids as a probe of protein dynamics. *Protein Sci.* **18**, 2603–2614 (2009). 19827094 10.1002/pro.264 PMC2821278

[41] Jankowski, M. et al. Ultrabroadband nonlinear optics in nanophotonic periodically poled lithium niobate waveguides. *Optica* **7**, 40–46 (2020).

[42] Chen, P. K. et al. Adapted poling to break the nonlinear efficiency limit in nanophotonic lithium niobate waveguides. *Nat. Nanotechnol.* **19**, 44–50 (2024). 37884657 10.1038/s41565-023-01525-w

[43] Zhou, B. B. et al. Parametrically tunable soliton-induced resonant radiation by three-wave mixing. *Phys. Rev. Lett.* **118**, 143901 (2017). 28430470 10.1103/PhysRevLett.118.143901

[44] Bizot, R. et al. All-fiber supercontinuum absorption spectroscopy for mid-infrared gas sensing. *APL Photonics* **9**, 111303 (2024).

[45] Jha, R. K. Non-dispersive infrared gas sensing technology: a review. *IEEE Sens. J.* **22**, 6–15 (2022).

[46] Wang, J. X. et al. On the adsorption characteristics and mechanism of methylene blue by ball mill modified biochar. *Sci. Rep.* **13**, 21174 (2023). 38040771 10.1038/s41598-023-48373-1 PMC10692330

[47] Fernández-Pérez, A. & Marbán, G. Visible light spectroscopic analysis of methylene blue in water; what comes after dimer? *ACS Omega* **5**, 29801–29815 (2020). 33251415 10.1021/acsomega.0c03830 PMC7689667

[48] Gordon, I. E. et al. The HITRAN2020 molecular spectroscopic database. *J. Quant. Spectrosc. Radiat. Transf.* **277**, 107949 (2022).

[49] Ding, Z. H. et al. Multi-object silicon photonic spectrometer. *Laser Photonics Rev.* **19**, 2400671 (2025).

[50] Grassani, D. et al. Mid infrared gas spectroscopy using efficient fiber laser driven photonic chip-based supercontinuum. *Nat. Commun.* **10**, 1553 (2019). 30948726 10.1038/s41467-019-09590-3 PMC6449389

[51] Lafforgue, C. et al. Broadband supercontinuum generation in nitrogen-rich silicon nitride waveguides using a 300 mm industrial platform. *Photonics Res.* **8**, 352 (2020).

[52] Yu, M. J. et al. Coherent two-octave-spanning supercontinuum generation in lithium-niobate waveguides. *Opt. Lett.* **44**, 1222–1225 (2019). 30821753 10.1364/OL.44.001222

[53] Sollapur, R. et al. Resonance-enhanced multi-octave supercontinuum generation in antiresonant hollow-core fibers. *Light Sci. Appl.* **6**, e17124 (2017). 30167225 10.1038/lsa.2017.124 PMC6062021

[54] MacDonald, R. L. & Lawandy, N. M. Efficient second-harmonic generation into the UV by using optically encoded silicate glasses. *Opt. Lett.* **18**, 595–597 (1993). 19802211 10.1364/ol.18.000595

[55] Travers, J. C. et al. High-energy pulse self-compression and ultraviolet generation through soliton dynamics in hollow capillary fibres. *Nat. Photonics* **13**, 547–554 (2019).

[56] Kröckel, L. & Schmidt, M. A. Extinction properties of ultrapure water down to deep ultraviolet wavelengths. *Optical Mater. Express* **4**, 1932–1942 (2014).

[57] Parriaux, A., Hammani, K. & Millot, G. Electro-optic frequency combs. *Adv. Opt. Photonics* **12**, 223–287 (2020).

[58] Ycas, G. et al. High-coherence mid-infrared dual-comb spectroscopy spanning 2.6 to 5.2 μm. *Nat. Photonics* **12**, 202–208 (2018).

[59] Nader, N. et al. Versatile silicon-waveguide supercontinuum for coherent mid-infrared spectroscopy. *APL Photonics* **3**, 036102 (2018).

[60] Di, Y. F. et al. Dual-comb spectroscopy from the ultraviolet to mid-infrared region based on high-order harmonic generation. *Photonics Res.* **11**, 1373–1381 (2023).

[61] Liu, X. Y. et al. Ultra-broadband and low-loss edge coupler for highly efficient second harmonic generation in thin-film lithium niobate. *Adv. Photonics Nexus* **1**, 016001 (2022).

[62] Conforti, M., Baronio, F. & De Angelis, C. Nonlinear envelope equation for broadband optical pulses in quadratic media. *Phys. Rev. A* **81**, 053841 (2010).

[63] Baronio, F. et al. Second and third order susceptibilities mixing for supercontinuum generation and shaping. *Optical Fiber Technol.* **18**, 283–289 (2012).

[64] Hansson, T. et al. Single envelope equation modeling of multi-octave comb arrays in microresonators with quadratic and cubic nonlinearities. *J. Optical Soc. Am. B* **33**, 1207–1215 (2016).

[65] Xiong, H. Z. et al. Proposal of on-chip ultrabroad supercontinuum generation ranging from visible to mid-infrared spectrum. *J. Lightwave Technol.* **42**, 4892–4898 (2024).

