# Supplementary material for: Ultraviolet-C to mid-infrared supercontinuum generation in periodically poled lithium tantalate waveguides
Source: Light Sci Appl. 2026 May 26;15:253. doi: 10.1038/s41377-026-02323-4 (PMC13212705; doi:10.1038/s41377-026-02323-4)
Supplement: Supplementary file 1 — Supplementary Materials for Ultraviolet-C to Mid-infrared Supercontinuum Generation in Periodically Poled Lithium Tantalate Waveguides [file 41377_2026_2323_MOESM1_ESM.docx]

Supplementary Information for

Ultraviolet-C to Mid-infrared Supercontinuum Generation in Periodically Poled Lithium Tantalate Waveguides

Hongzhi Xiong,^1†^ Xinmin Yao, ^1†^Ming Zhang, ^1,3^* Qingrui Yao, ^1^ Huan Li, ^1^ Zejie Yu, ^1^ Gong Zhang, ^1^ Liu Liu, ^1^ Yaocheng Shi,^1^ Hon-ki Tsang ^4^, and Daoxin Dai, ^1,2,3^*

1 State Key Laboratory for Extreme Photonics and Instrumentation, Zhejiang Key Laboratory of Optoelectronic Information Technology, College of Optical Science and Engineering, International Research Center for Advanced Photonics, Zhejiang University, Zijingang Campus, Hangzhou 310058, China;

2 Intelligent Optics and Photonics Research Center, Jiaxing Research Institute, Zhejiang University, Jiaxing 314000, China;

3 Ningbo Global Innovation Center, Zhejiang University, Ningbo 315100, China

4 Department of Electronic Engineering, The Chinese University of Hong Kong, Hong Kong SAR, China.

†: These authors contributed equally to this work.

* Corresponding author. Email: mingzhang@zju.edu.cn, [dxdai@zju.edu.cn](mailto:dxdai@zju.edu.cn)

1. **The simulations of electric fields.**

In Supplemental Fig. S1, we calculate the normalized electric fields for the TE_0_ mode in both the *W*=1.3 μm and *W*=3 μm waveguides, using Finite Difference Eigenmode (FDE) methods. At 3800 nm wavelengths, the *W*=1.3 μm the waveguide fails to effectively confine the mode. At 280 nm, the mode area is roughly twice that of *W*=1.3 μm waveguide, thereby reducing the nonlinear interaction at these wavelengths.


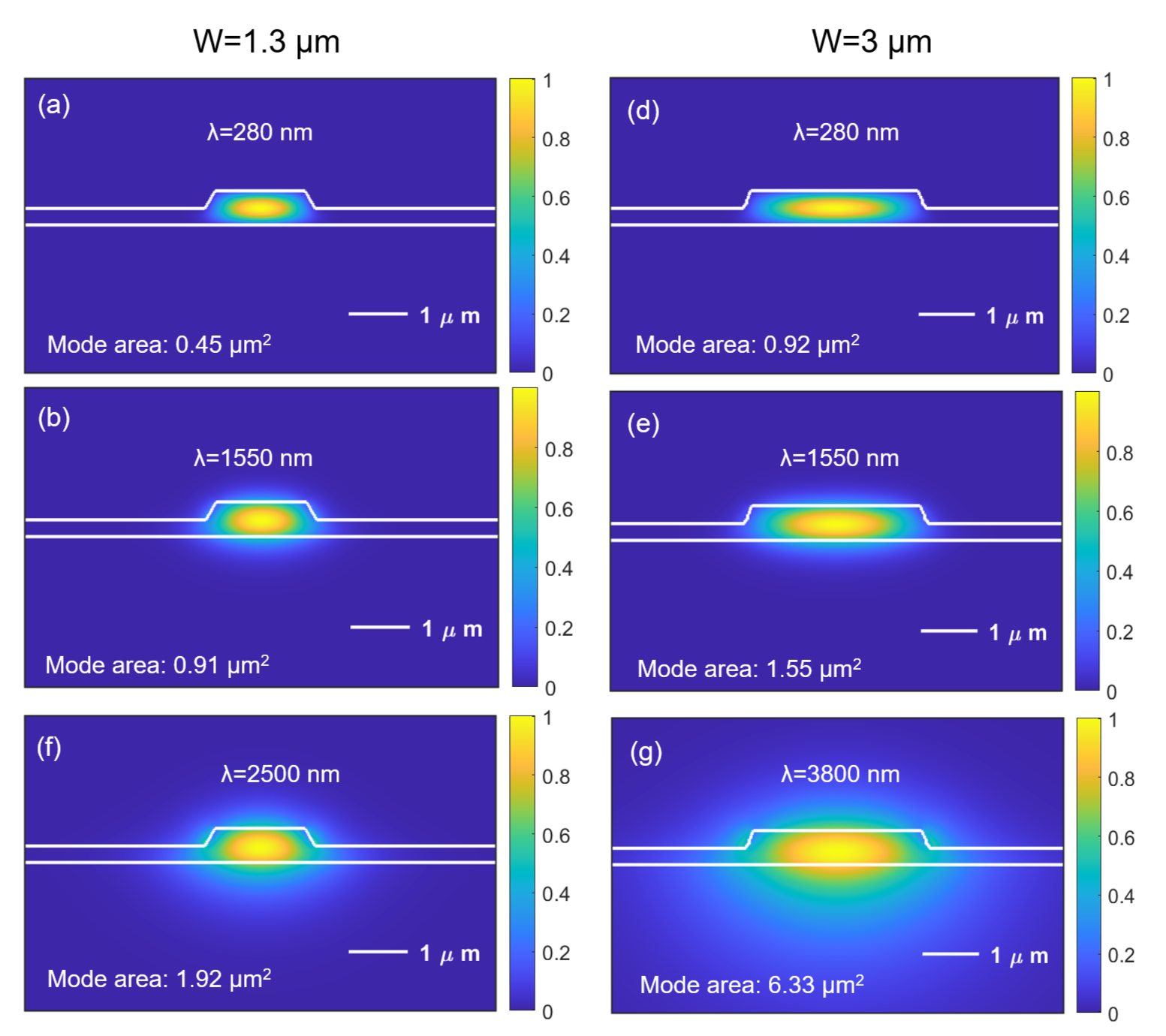


**Fig.S1.** On the left side, the normalized electric fields for the TE_0_ mode are shown for a top width of *W*=1.3 µm at wavelengths of 280 nm (a), 1550 nm (b), and 2500 nm (c), respectively. On the right side, the normalized electric fields for the TE_0_ mode are shown for a top width of *W*=3 µm at wavelengths of 280 nm (d), 1550 nm (e), and 3800 nm (f), respectively.

1. **Theoretical calculations of the impact of internal pulse energy.**

**In Supplementary Fig. S2, we present the calculated spectral intensity as a function of the on-chip average pump power across different wavelengths. In this simulation, a 100 fs pulse at 1550 nm is used, and the average pump power is varied from 0 to 30 mW. As shown in the figure, when the on-chip average pump power increases from 0 to 10 mW, the supercontinuum bandwidth expands significantly. However, when the pump power exceeds 10 mW, the bandwidth exhibits much weaker further broadening. Therefore, the maximum output power of the femtosecond laser used in our experiment is chosen as 100 mW, corresponding to an on-chip average power of ~10 mW regarding the fiber-chip coupling losses.**

**
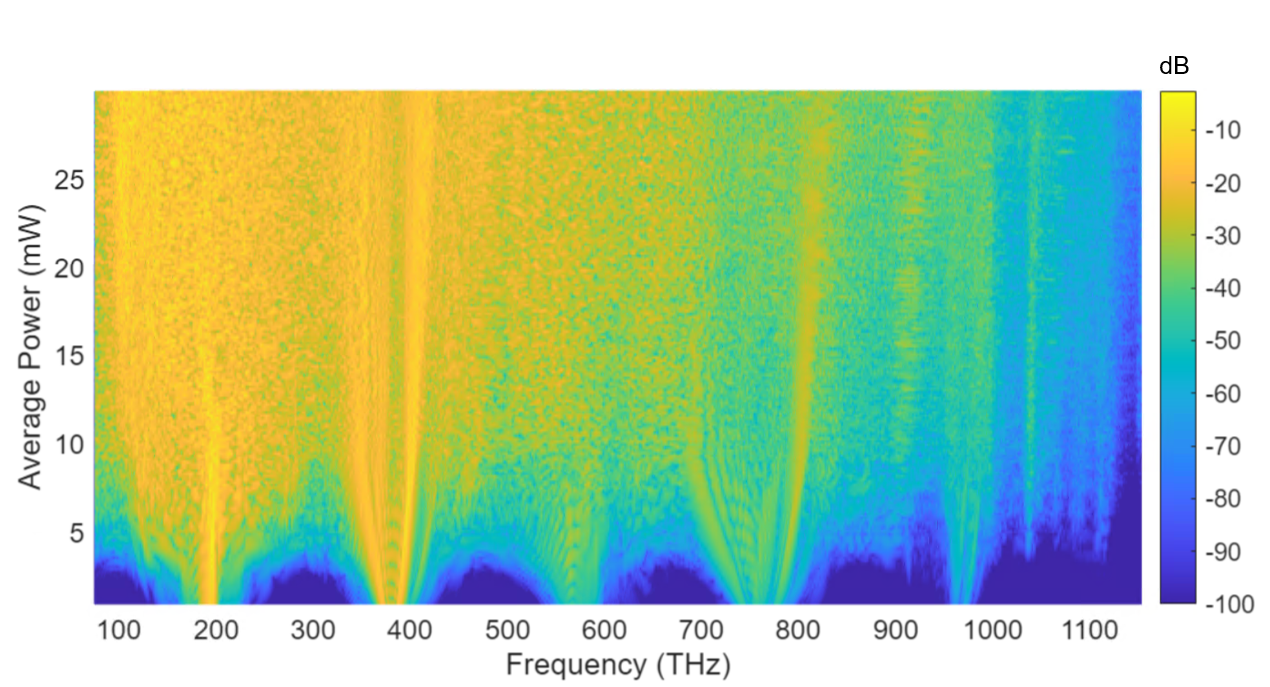
**

**Fig.S2. Calculated spectral intensity as a function of the on-chip average pump power across different wavelengths. In this simulation, a 100 fs pulse at 1550 nm is used, and the average pump power is varied from 0 to 30 mW.**

1. **The comparison of effective refractive indices for the eigenmodes of lithium niobate (LN) and lithium tantalate (LT).**

We simulate the effective refractive indices for the eigenmodes of LN and LT. In Supplemental Fig. S3a, we calculate the effective refractive indices for the eigenmodes in 600 nm height, 1.2 µm wide, 400 nm etch depth LN waveguides in the x-z plane at different wavelengths for a large bending radius of *R* = 100 µm. The mode hybridization for the generated Supercontinuum at TE_0_ mode occurs around 900 nm with TM_1_ and at 1350 nm with TM_0_. In Supplemental Fig. S3b, we calculate the effective refractive indices for the eigenmodes in LT waveguides in the x-z plane with the same bending radius and waveguide cross-section，and no mode hybridization occurs for TE_0_ across such a large wavelength range, and the TE_0_ mode consistently has the highest effective index. In Supplemental Fig. S3c, we calculate the effective refractive indices for the eigenmodes in LT waveguides in the x-z plane with a small bending radius of *R* = 60 µm from 400 nm to 1800 nm, and no mode hybridization occurs for TE_0_ across such a large wavelength range.


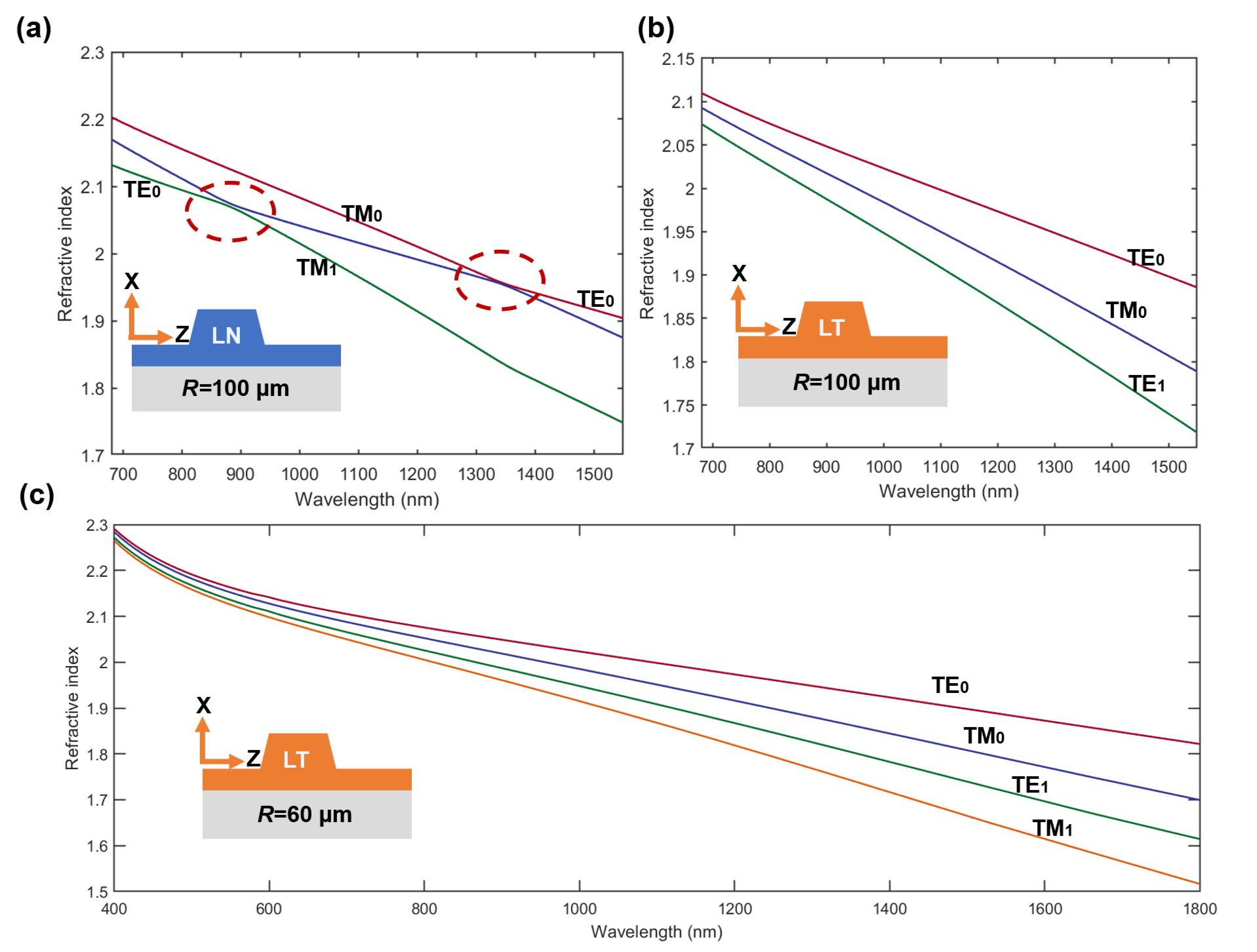


**Fig. S3.** (a) Effective refractive indices of the eigenmodes in 600 nm height, 1.2 µm wide, 400 nm etch depth LN waveguides in the x-z plane for a large bending radius of *R*=100 µm. (b) Effective refractive indices of the eigenmodes in LT waveguides with the same structural parameters as in (a), also in the x-z plane with a bending radius of *R*=100 µm. (c) Effective refractive indices of the eigenmodes in LT waveguides with the same structural parameters as in (a), but with a smaller bending radius of *R* = 60 µm from 400 nm to 1800 nm.

1. **Discussion about the generated modes.**

While the periodic poling scheme is designed for phase matching of the TE fundamental (TE_0_) mode, quasi-phase matching of higher-order modes may still occur in the waveguide. Here, we provide an analysis of this phenomenon.

First, the pump light used in the experiment is with the TE_0_ mode, and the PPLT waveguide is designed as a smooth taper, ensuring that modal conversion caused by structural abrupt variations is negligible. Second, numerical simulations show that the waveguide supports only the TE_0_ mode for the wavelength above 1900 nm, confirming that the supercontinuum in the long wavelength range is strictly single-mode. Third, in the short-wavelength region, although a PPLT waveguide can achieve quasi-phase matching between the fundamental and higher-order modes in theory, the conversion efficiency of these higher-order interactions is significantly lower than that of the TE_0_ mode and thus the generated higher-order modes are negligible.

Here, we used Lumerical Mode to calculate the modal fields and conversion efficiencies for second-harmonic generation (SHG) around the central wavelength range of 275–320 nm, as analyzed in Fig. S4. The conversion efficiency, denoted as η, is defined in the same manner as that in previous works^1, 2^. The results indicate that the conversion efficiency of the TE_0_ mode at 280 nm is approximately one order of magnitude higher than that of the TE₂ mode, while the TE₁ mode generation can be neglected. This behavior arises because the effective cross section between the higher-order modes and the fundamental mode is significantly smaller than that of the TE_0_ mode. Therefore, although quasi-phase-matched SHG involving the TE_0_–TE₁ or TE_0_–TE₂ mode interactions can occur, their output power is much lower than that of the TE_0_–TE_0_ SHG process.

Finally, for applications requiring strict single-mode operation, the mode purity can be further enhanced by coupling the supercontinuum output into a short single-mode waveguide section or collecting the light with a single-mode fiber. In this work, we used PM1550 single-mode fibers and UV single-mode fibers (SM300-SC) for coupling in the NIR–MIR and UV–visible ranges, respectively, making sure that the SC generation are single-mode.


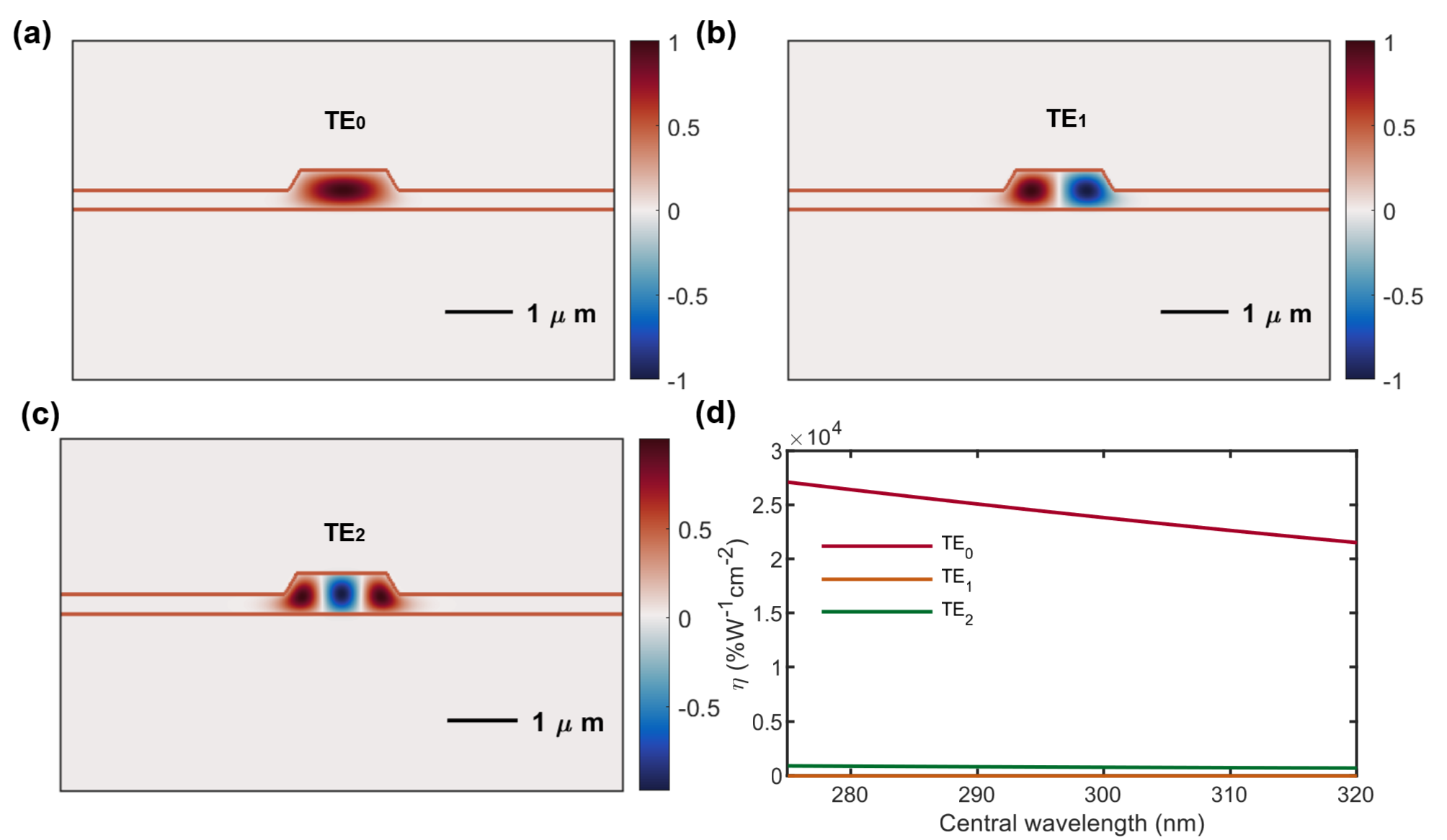


**Fig. S4.** Normalized electric fields for different modes of TE_0_ (a), TE_1_ (b), and TE_2_ (c) at the wavelength of 280 nm. (d) Simulated conversion efficiency of different second harmonic modes at the central wavelength from 275 nm to 320 nm.

1. **The phase mismatch in the PPLT waveguide.**

For the proposed chirped PPLT supercontinuum waveguide, Section I is designed for cascaded χ²-based SCG to achieve NIR output, where the performance is strongly influenced by the quasi-phase-matching condition. The phase mismatch at the central wavelength of the PPLT waveguide is expressed as

$$\Delta\beta=\beta_{2\omega}-2\beta_{\omega}-\frac{2\pi}{\Lambda}$$

where $\beta_{2\omega}$and $\beta_{\omega}$are the propagation constants of the second-harmonic and fundamental waves, respectively, and $\Lambda$is the period of the poling waveguide. This parameter quantifies the degree of phase matching between different frequencies (or modes). When $\Delta\beta=0$, one has the perfect phase matching. Otherwise ($\Delta\beta\neq0$), the mismatch leads to a reduction in conversion efficiency.

In Fig. 2d, even though we assumed a poling period of 4.13 μm to approximately satisfy the condition of $\Delta\beta=0$at the pump’s central wavelength, the deviations of the waveguide dimensions such as the etching depth, the width, and the film thickness can introduce additional phase mismatch. To more accurately reproduce the experimental results, an extra $\Delta\beta$term was incorporated in the simulation. Fig. S5 presents the simulated results for the cases with different $\Delta\beta$. As $\Delta\beta$approaches zero more, the quasi-phase-matching becomes more excellent, resulting in a broader and flatter supercontinuum spectrum.

In practice, such phase mismatches can be mitigated effectively through adaptive poling techniques^3^, which allow precise measurement and compensation of the variation of the LT thin-film thickness, thereby achieving $\Delta\beta=0$and further improving the performance of the supercontinuum.


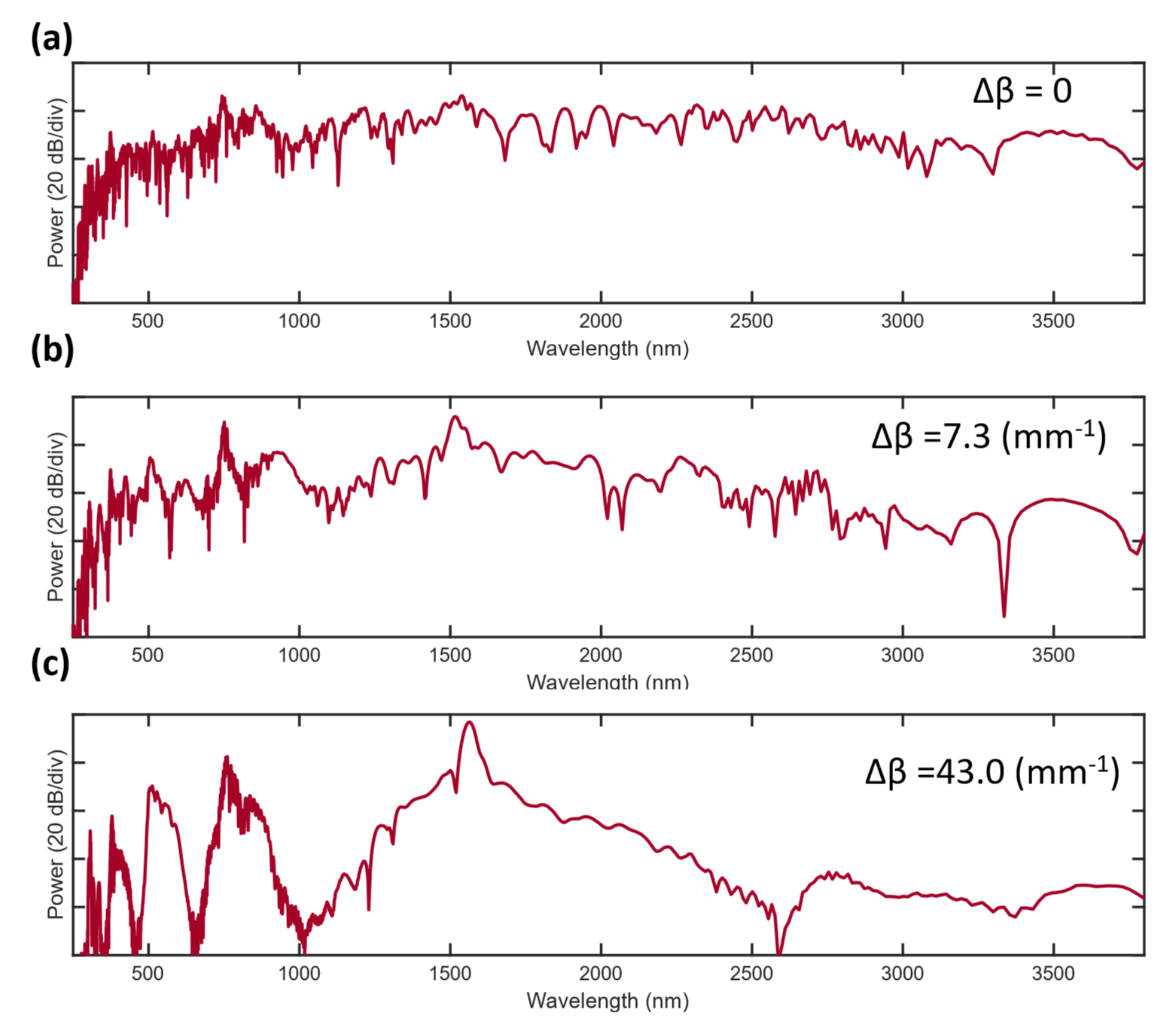


**Fig. S5.** Simulated results of our proposed PPLT waveguide for the cases with different $\Delta\beta$. It can be seen that as $\Delta\beta$approaches zero more, the quasi-phase-matching becomes more excellent, resulting in a broader and flatter supercontinuum spectrum.

1. **The DFG performance in chirped PPLT.**

Fig. S6a shows the top-views compare the complete waveguide (whose structure is identical to the design presented in the main text) with a truncated waveguide derived from the complete structure by removing Section III while keeping all other parameters unchanged. The numerical simulations results shown in Fig. S6b indicate that, within the 3300–3800 nm wavelength range, the complete waveguide (consisting of Sections I–III) exhibits significant enhancement relative to the truncated waveguide without Section III.


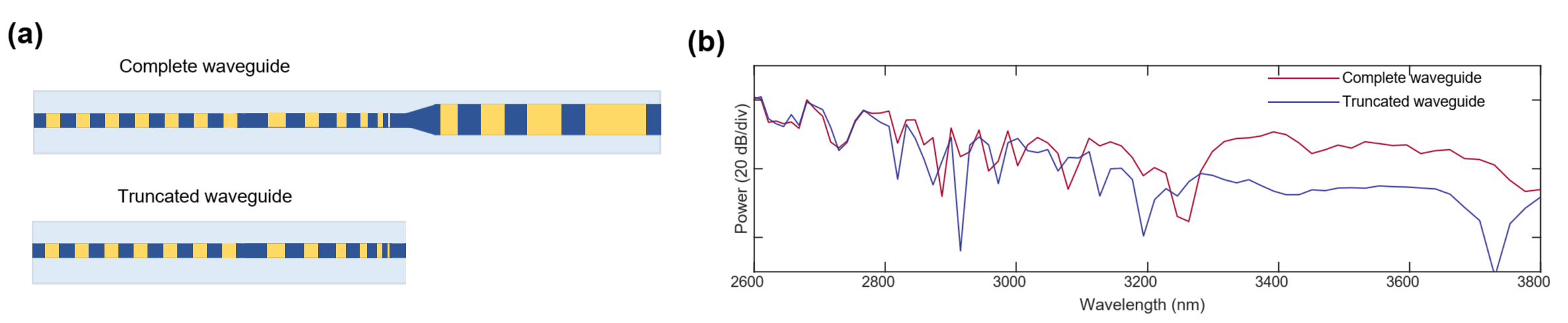


**Fig. S6.** (a) The top-views of the complete waveguide (whose structure is identical to the design presented in the main text) and a truncated waveguide (whose structure is derived from the complete structure by removing Section III while keeping all other parameters unchanged). (b) The numerical simulation results to compare the two types of waveguides.

1. **The temperature stability test of chirped PPLT.**

Fig. S7 shows the measured output spectra of the chirped PPLT waveguide when the temperature was set at 25 °C, 35 °C, and 45 °C, respectively. As it can be seen, the present SC source operates reliably without any spectral discontinuities as the temperature varies from 25 °C to 45 °C, while there were some variations observed from the measured spectra. Such spectral variations are mainly due to the temperature-induced change of the waveguide’s effective refractive index, which results in the change of the dispersion and the quasi-phase-matching condition. Therefore, it is helpful to introduce precise temperature control for the chirped PPLT when used for sensing applications.


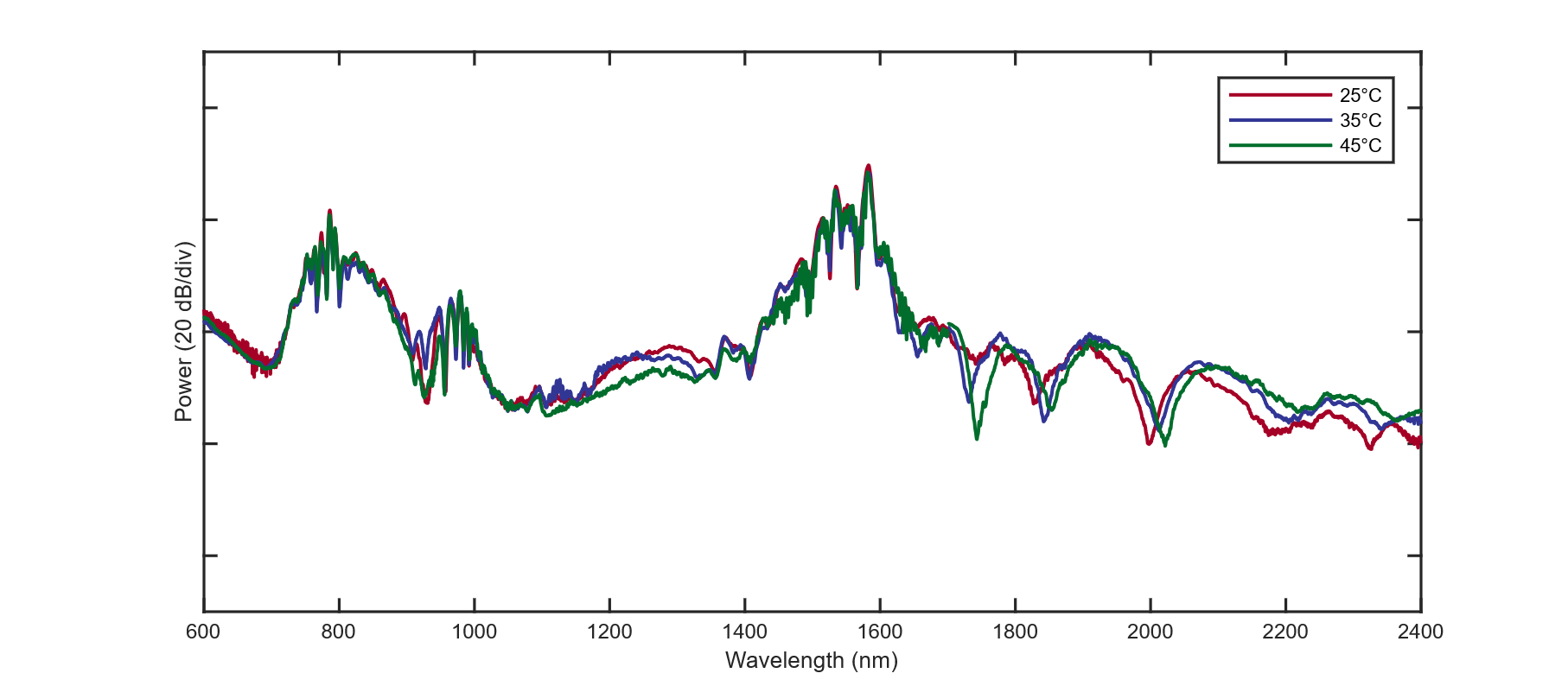


**Fig. S7.** Measured spectra of the chirped PPLT when operating at different temperatures of 25 °C, 35 °C, and 45 °C.

1. **Dispersion engineering with different etching depths.**

In addition to the waveguide width, the etching depth also greatly affects the dispersion of the waveguide. Figure S8 shows the phase mismatch, dispersion, and group velocity for etching depths of 400 nm and 500 nm. As the etching depth increases, the dispersion of the supercontinuum waveguide shifts further toward the anomalous-dispersion regime, and the dispersive-wave wavelength moves farther away from the pump. It is worth noting that, compared with supercontinuum waveguides relying on third-order nonlinearities, chirped PPLT waveguides impose less requirements on the dispersion control, because the chirped PPLT enables the second-order nonlinear wavelength conversion through quasi-phase matching by changing the poling period, offering high design flexibility. In contrast, the conversion efficiency for a certain wavelength range of conventional third-order nonlinear supercontinuum waveguides is highly determined by the waveguide dispersion.


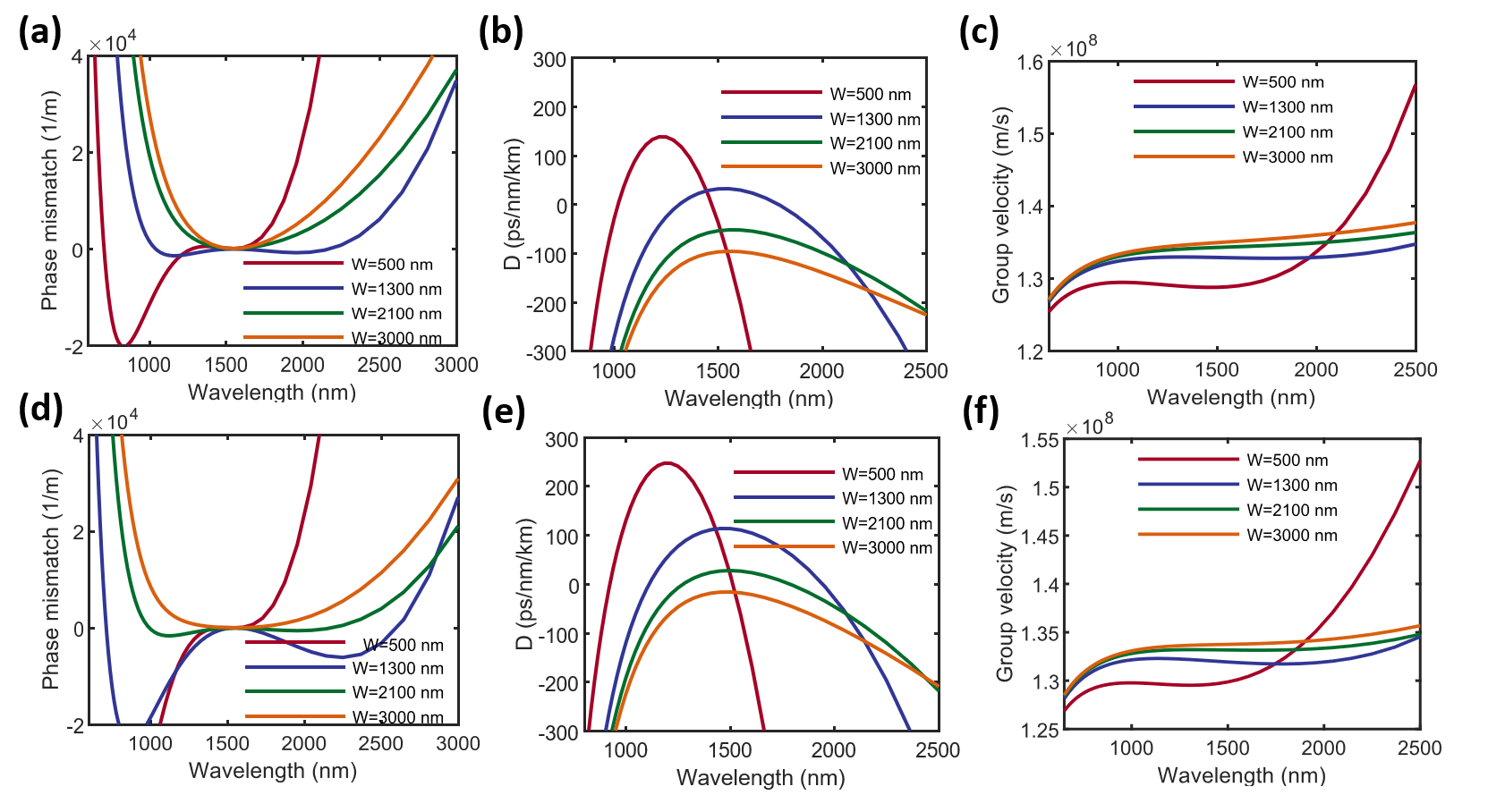


**Fig. S8.** (a), (b) and (c): The phase mismatch, dispersion and group velocity of LT-on-insulator waveguides with height=600 nm and etch depth=400 nm and at different widths of W=500, 1300, 2100 and 3000 nm, respectively. (d), (e) and (f): The phase mismatch, dispersion and group velocity of LT-on-insulator waveguides with height=600 nm and etch depth=500 nm and at different widths of W=500, 1300, 2100 and 3000 nm, respectively.

1. **Three-Wave Mixing Processes in Chirped PPLT.**

Here we calculate the phase-matching wavelengths as a function of the poling period, as shown in Fig. S9. The SFG and DFG curves are obtained from Eqs. (1) and (2) in the Materials and Methods. These curves allow us to approximately estimate the quasi-phase-matched wavelengths for supercontinuum generation in PPLT waveguides. The quasi-phase-matching wavelength decreases monotonically with decreasing poling period, which is consistent with the 3WM process under CW pumping. It is worth noting that, based on our numerical simulation, the real spectral bandwidth of the generated supercontinuum is typically slightly broader than that predicted by Eqs. (1) and (2), which is expected because the femtosecond pump possesses an intrinsically broad spectrum.

To enable supercontinuum generation from the visible to the deep-UV, we implement a linearly decreasing poling period in the waveguide Section II so that quasi-phase matching can be satisfied from near-infrared to UV. The minimum poling period is 0.95 µm, corresponding to the SFG QPM wavelength at around 290 nm. This value is largely constrained by the fabrication limits of our poling process. Even shorter wavelengths may in principle be achieved using smaller poling periods, while the fabrication with ultrashort periods in X-cut LT wafer remains extremely challenging. In Section III, we employ a linearly increasing poling period to achieve DFG-based quasi-phase matching in the mid-infrared region.


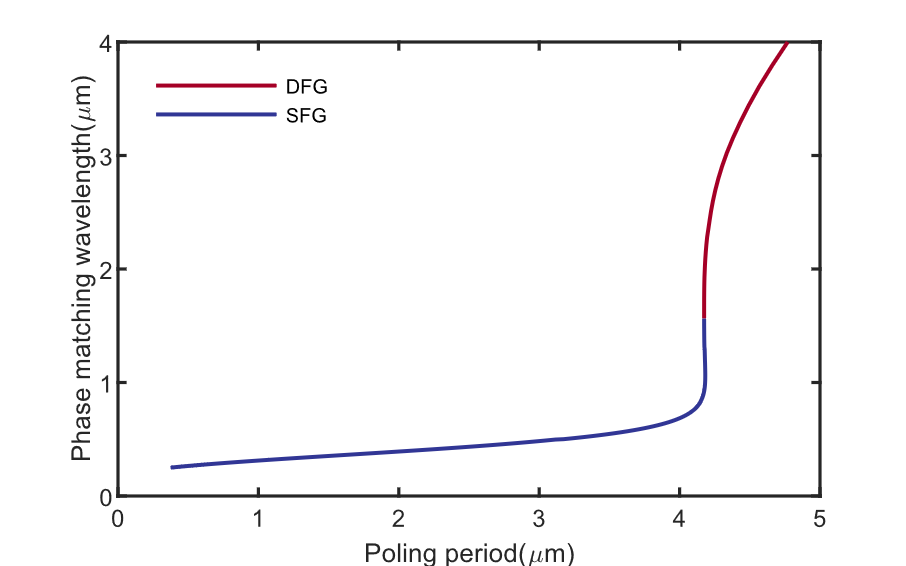


**Fig. S9.** Calculated phase matching wavelength as a function of poling period.

Next, we present the numerical simulation results for structures with minimum poling periods of 0.95, 3, and 2 µm, while keeping all other parameters identical to those used in the main-text design (see Fig. S10). As expected, decreasing the minimum poling period shifts the short-wavelength cutoff of the supercontinuum toward the UV. The obtained bandwidths are also generally in good agreement with the predictions from Fig. S9.


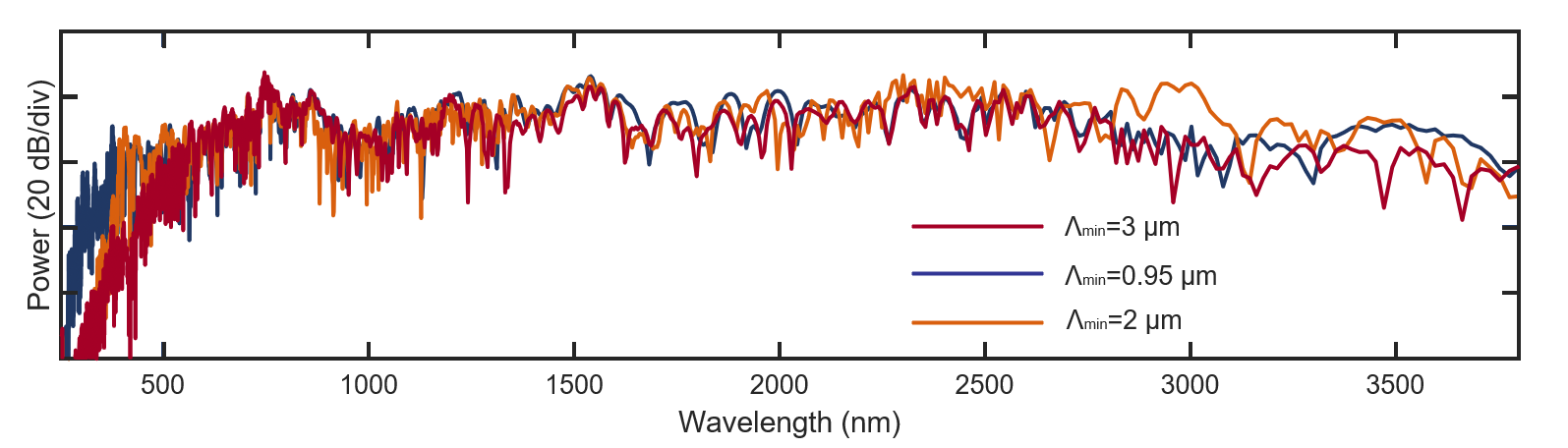


**Fig. S10.** Numerical simulation results for structures with minimum poling periods of 0.95, 3, and 2 µm, while keeping all other parameters identical to those used in the main-text design.

**References:**

[1] J. Zhao *et al.*, “Shallow-etched thin-film lithium niobate waveguides for highly-efficient second-harmonic generation,” Opt Express*,* 28(13), 19669-19682 (2020).

[2] C. Wang *et al.*, “Ultrahigh-efficiency wavelength conversion in nanophotonic periodically poled lithium niobate waveguides,” Optica*,* 5(11), (2018).

[3] P.-K. Chen *et al.*, “Adapted poling to break the nonlinear efficiency limit in nanophotonic lithium niobate waveguides,” Nature Nanotechnology, (2023).
